# Supplementary material for: Improved pore structure characterization and classification of strong diagenesis sandstones by data-mining analytics in Tazhong area, Tarim Basin
Source: PLoS One. 2024 Aug 27;19(8):e0309092. doi: 10.1371/journal.pone.0309092 (PMC11349092; doi:10.1371/journal.pone.0309092)
Supplement: Attachment — Submitted filename: Revision Note V3FIN.docx [file pone.0309092.s002.docx]

Dear Editors and Reviewers:

Thank you for your letter and for the reviewer comments concerning our manuscript entitled “Improved Pore Structure Characterization and Classification of Strong Diagenesis Sandstones by Data-mining Analytics in Tazhong Area, Tarim Basin”. Those comments are all valuable and very helpful for revising and improving our paper, as well as the important guiding significance to our successive researches. Comments are carefully studied and have made correction which we hope meet with approval. **Revised portion are marked in yellow in the paper.**

The main corrections in the paper and the responds to the reviewer’s comments are as following:

Responds to the reviewer’s comments:

**Reviewer #1:**

Thanks for your comments on our paper very much. It is a great encouragement to our research. The majority of manuscript is revised according by your complete comments and your comments bring us lot thinking on further research. The revisions are mainly focused on following aspects.

**1. Response to comment: Petrology characteristics of sandstone should be moved to the results part, it is your own research results, right? And the sample number of sandstone should be added in Fig.2 and Fig.3.**

Response:

Thank you for pointing out the oversight. As the reviewer's valuable suggestion, we have made the necessary adjustments to our manuscript. The petrographic characteristics of the sandstone samples have been relocated to the Results section, where they are more appropriately discussed. Additionally, we have included the number of sandstone samples in both Figure 2 and Figure 3, ensuring a more comprehensive presentation of our findings. Thank you for the suggestion.

**2. A and B of Fig, 3 did not include Folk (1973) sandstone classification, what’s the figure captions of c and d, which should include sample data, depth, PPL or XPL or CL.**

Response:

We appreciate the reviewer's feedback and have made the necessary revisions to the citation of A and B of Fig, 4. Additionally, we have changed the name of Figure 3 accordingly. Thank you for the suggestion.

**3. Line 84, X-ray diffraction mentioned, where is your XRD data?**

Response：

It has been published in previous study in *Journal of Applied Geophysics*, so we had added in the reference.

**4. Response to comment: The author combined the methodology and results together, it is confusing. The analysis and testing methods and quantities should be put together.**

Response:

As your recommendation, we have now separated the research methodology and results sections, allowing for a clearer distinction between the two. Thank you for the suggestion.

**5. Response to comment: The whole manuscript is actually based on the classification of sandstones using thin sections and mercury intrusion, but I do not understand the roles of ‘strong diagenesis’ in it, what’s impact of strong diagenesis on the formation of sandstone.**

**Response:**

We have made the necessary additions in our manuscript, specifically on line 359-396, and in Table 5, it illustrated that for Type Ⅲ to Ⅵ, it reflects strong diagenesis effect on the pore structure, Where we have emphasized the significant role of diagenesis in the formation process of sandstone. Thank you for the suggestion.

**6. Response to comment: What is MICP? I did not see the full name of it in the text.**

**Response：**

In accordance with the reviewer's suggestion, we have now included the full names of the abbreviations upon their first occurrence in the text and we have compiled all abbreviations and their corresponding full forms into a table, which is included in the Introduction section. Thank you for the suggestion.

**7. Response to comment: What is the function of cathode luminescence in the pore structure classification?**

Thank for you suggestion, We have made the necessary additions in our manuscript on line 115-120.

**Reviewer #2:**

Thanks for your comments on our paper very much. It is a great encouragement to our research. The majority of manuscript is revised according by your complete comments and your comments bring us lot thinking on further research. The revisions are mainly focused on following aspects.

**1. Response to comment: The abstract of the manuscript is very detailed. However, sometimes such abstract cannot clearly reflect the key insights and particularities of the research. Therefore, it is suggested that the authors optimize the structure of the abstract to make it more concise.**

Response:

We greatly appreciate the reviewer's valuable feedback regarding the abstract section of our manuscript. Taking their suggestion into consideration, we have carefully revised and condensed the abstract to ensure its conciseness and clarity. This modification will provide readers with a more succinct summary of our research findings. Thank you for the suggestion.

**2. Response to comment: References should be supplemented with the latest research results. The introduction section lacks support of recent years. It is suggested that authors update references. Due to the lack of support from relevant references, it is impossible to determine whether the author has tracked the latest progress, which may lead to problems in summarizing the current situation and sorting out the problems. It is very important for readers to understand the research background.**

Response: Thank for you suggestion, We have added the recent reference in our manuscript on line 50-51.

**3. Response to comment: The text in Figure 1 should be in Times New Roman font. This Figure can be further optimized. The scale in Figure 3 cannot be seen clearly. Part of the text in Figure 6 is too small to read. Fig.8 is not clear, and the content cannot be seen clearly. Some text sizes in Figure 10 exceed the main text. They're lacks of explaining the Figure 11. please describe more.**

Response:

After checking all figures carefully，Our files have met the rules for PLOS ONE journal. Here below the rules. Thank you for your suggestion.

**4. Response to comment: Tables 2 and 5 have inserted figures in the table. Perhaps it would be more appropriate to directly modify it to a highly summarized figure. The authors can consider whether modifications are needed (this is only a suggestion)**

Response: Thank you for your suggestion.

**5. Response to comment: Please further revise the statements in the paper that are difficult to read. Especially the naming of some new classifications. Part of the paper's discussion is not very user-friendly to readers and can be further optimized for expression. Commonly the abbreviations are explained the first time of the appearance of the corresponding terms. Here the abbreviations MICP, PCA, LDA, appearing in abstract, should be attributed to specific terms.**

Response:

In accordance with the reviewer's suggestion, we have now included the full names of the abbreviations upon their first occurrence in the text and we have compiled all abbreviations and their corresponding full forms into a table, which is now included in the Introduction section. Thank you for the suggestion.

**6.Response to comment:** **Please add more description of the new findings and significance of research. Especially in the abstract and conclusion section.**

Response: we have added it in the abstract and conclusion.
