# Supplementary material for: Improved pore structure characterization and classification of strong diagenesis sandstones by data-mining analytics in Tazhong area, Tarim Basin
Source: PLoS One. 2024 Aug 27;19(8):e0309092. doi: 10.1371/journal.pone.0309092 (PMC11349092; doi:10.1371/journal.pone.0309092)
Supplement: Attachment — Submitted filename: TF_Responds to the reviewer.plosone.docx [file pone.0309092.s003.docx]

Responds to the reviewer’s comments:

**Reviewer #3:**

Thanks for your comments on our paper very much. It is a great encouragement to our research. The majority of manuscript is revised according by your complete comments and your comments bring us lot thinking on further research. The revisions are mainly focused on following aspects.

**1. Response to comment: Introduction: Author should introduce some new research trend on the characterization and classification of pore structure in tight sandstone reservoirs. Besides, it is necessary to clarify the importance of pore structure in tight sandstone.**

Response:

We apologize for the oversight and have duly included the current research trend in the characterization and classification of pore structure in tight sandstone reservoirs in lines 33-36 as per your suggestion. Furthermore, we agree that it is essential to clarify the importance of pore structure in tight sandstone reservoirs, and have incorporated this in lines 31-33 as advised. Thank you for your valuable input.

**2. Response to comment: Geological setting. Author should add some details and caption on each picture in the Fig.1 Where is the TZ11 and TZ12?.**

Response:

We strongly agree with the reviewer suggestion and we have added some details to Fig.1 to illustrate the location of TZ11 and TZ12 wellblock. Thank you for your suggestion.

**3. Response to comment: Geological setting. Line 65 what is “It” mean?**

Response:

In this context, "it" refers to the stratigraphic formation, and we acknowledge that it was our oversight not to express this clearly. We have made the necessary adjustment by replacing "it" with "The stratigraphic formation" to improve clarity in the expression in line 74. Thank you for pointing this out.

**4. Response to comment: Geological setting. Line 65-68. The lithological characteristics of Silurian system should be clarified carefully in the text.**

Response:

We greatly appreciate the reviewer's suggestion and have incorporated a detailed description of the lithological characteristics of the Silurian strata in lines 76-83. Thank you for the suggestions.

**5. Response to comment: Geological setting. Line 68-line 70: Some references are needed to support the sedimentary facies.**

Response:

We are referencing the sedimentary facies conclusions from the literature; However, due to our oversight, we neglected to cite the references. We have now added the citations in the text to support the sedimentary facies in lines 86-87.Thank you for the suggestion.

**6. Response to comment: Methodology .Author should explain the experimental analysis (e.g. MICP) or the source of experimental data.**
Response:

We fully agree with the reviewer's feedback and have included a section explaining the analysis of the mercury injection capillary pressure (MICP) experiments, along with clarifying the source of the experimental data in lines 92-99.Thank you for the suggestion.

**7. Response to comment: Petrology characteristics of sandstone. This part is too simple and confusing. There is no caption and annotation in the Fig.5. The petrology characteristics of sandstone may be different in different Formation or member.**
Response:

We apologize for not labeling clearly, the appropriate caption have been added. The explanation for Figure.5 is presented below in Lines 145-147. The title of Figure 5 has been expanded to include detailed annotations. The study zone for this research in this section focuses on the Upper-3rd submember of the upper Kepingtag member, so we don’t pay attention to the comparison with other submembers. It can be observed from Figure.5 that the petrology characteristics of the sandstone are consistent within this submember. Thank you for the suggestion.

**8. Response to comment: There is no discussion part in the manuscript. The structure of the manuscript needs to be reorganized.**
Response:

It was an oversight on our part not to label which section is the discussion. In this paper, we have integrated the discussion and results. For instance, in section 4.3, we discuss the relative independence of the information about the impact of slope in the scree plot on various features. Similarly, in section 4.4, we discuss the distribution trends of Pd pore-throat pressures and the selectivity trend distributions, among others. We have renamed the entire fourth section as "Results and Discussion." Thank you for the suggestion.

**9. Response to comment: There are many language issues in the manuscript. The level of English of your manuscript does not meet the international journal's standard.**

Response:

We have conducted a thorough review of the entire manuscript and addressed numerous language issues, such as those in lines 37-38,39-44,63-64,65-68,104-105,116-117,216-217 307-308 ,237-238 and 412-414 among others (marked in the text; not listed here). This has enhanced the English proficiency of the manuscript. We appreciate your feedback, which brought this to our attention, and we have made the necessary revisions based on your suggestions.

**Reviewer #4:**

Thanks for your comments on our paper very much. It is a great encouragement to our research. The majority of manuscript is revised according by your complete comments and your comments bring us lot thinking on further research. The revisions are mainly focused on following aspects.

**1. Response to comment: The use of “The Silurian system”and “Sedimentation-diagenesis controls” as keywords in this article is inappropriate. It is recommended that the author readjust the keywords of this article.**

Response:

We appreciate your suggestion, and upon careful consideration, we agree that using "Silurian system" and "sedimentary-diagenetic control" as keywords may not be appropriate. Accordingly, we have replaced these terms with "strong diagenesis sandstone" and "low-permeability reservoir" as per your recommendation. Thank you for your valuable input.

**2. Response to comment: In line 39, the author mentioned that analyzing MICP data does not reflect the real pore type, but the classification of pore structure in this article is based on MICP data. Does the MICP data analysis used in the article specifically adopt certain changes?**
Response:

We feel sorry for the previous words to make reviewers misunderstand. The essence of this article is to highlight that simple statistical description and regression analysis based on mercury injection capillary pressure (MICP) data often fall short in capturing the true pore types. This limitation stems from the inadequacy of overly simplistic models in effectively classifying pore structures and the significant impact of subjective human factors on the low efficiency of simple statistical descriptions, leading to inaccurate pore type characterization. In this study, we have employed an advanced approach with machine learning methods and models based on MICP data to achieve the classification and characterization of pore structures. This approach allows for a more comprehensive and accurate processing of data, minimizing the influence of subjective factors and enabling detailed analysis and classification of the data, thus facilitating precise classification and characterization of pore structures. Thank you for bringing this to our attention.

**3. Response to comment: In lines 42-44, the shortcomings of nuclear magnetic technology and CT imaging techniques are mentioned, but the author's expression is not clear.**

Response:

We have revised the discussion on the limitations of nuclear magnetic technology and CT imaging techniques in lines 48-52. Thank you for the suggestion.

**4. Response to comment: The lists of nomenclature in line 61, since the full names and their corresponding abbreviations have already been mentioned earlier, there is no need to place them separately in the table.**

Response:

Based on your suggestion, we acknowledge that the nomenclature table was indeed redundant, and we have removed it. Thank you for the suggestion.

**5. Response to comment: The Pd, Smin, and Rd in the text should be written as Pd, Smin, and Rd, expressed using subscripts; Mpa should be written as MPa, and many physical quantities mentioned in the text have obvious errors in their units.**

Response:

Thank you for bringing this to our attention. We have rectified the formatting issue, and now it is correctly written as P_d_,S_min_,R_d_ and MPa. Additionally, we have corrected the erroneous units of the physical quantities mentioned in the article. Thank you for the suggestion.

**6. Response to comment: In lines 107-108, it is mentioned that " It can be classified into four rock types ", but why are there five types of rocks in Figures 4 (a) and (b)? What components do Q, F, and R represent in Figure 5? Suggest providing an explanation.**

Response:

There are actually five rock types. It was our mistake to list them as four, and we have now made the necessary correction in line 131. Thank you for pointing that out. Q represents Quartz, F represents Feldspar, and R represents Rock Fragments. The QFR ternary plot is a graphical method used to describe the proportions of clastic particles in sedimentary rocks. In academic papers on sandstone reservoir studies, the sandstone classification ternary plot is widely used to differentiate sandstone types. We have now added the full name "QFR ternary plot" to Figure 5(a) and Figure 5(b) .Thank you for the suggestion.

**7. Response to comment:** **Placing XRD data in the reference list in lines 113-120 is deemed inappropriate, and it is recommended that the author revise it.**

Response:
It's inappropriate to include XRD data in the reference list as its conclusions are derived from previous studies. Therefore, we have removed the XRD data from this section and directly referenced the previous studies in line 138.Thank you for the suggestion.

**8. Response to comment:** **In Figure 8, the width of each column in the bar chart is different, and it is recommended to set it to a uniform width.**
Response:

There may be some confusion here, Fig. 8 is not a bar chart, and it is a cluster dendrogram that has already been standardized. Thank you very much for your suggestion.

**9. Response to comment: The use of references in this article is not standardized. The connected references in lines 41 and 42 should be placed in the same parentheses, and the same issue occurs in line 78; Lines 338-340 introduce the author's work and there is no need to add references.**
Response:

Thank you for pointing out the issue with the referencing format. We have now placed the linked references in the brackets in line 48, and the same issue has been rectified in Line 103 as well. We fully agree that the introduction of the author's work does not require the addition of references, and it has already been deleted. Thank you very much for your suggestion.

**10. Response to comment: The analysis and conclusion section of this article does not highlight the importance and innovation of the work done in the article.**
Response:

We fully agree with your opinion and indeed felt that the conclusion lacked some explanation regarding the importance and innovation of our work in Line 451-455. Following your advice, we have added emphasis on the importance and innovation in the conclusion. Thank you very much for your suggestion, which has improved the article.

**11. Response to comment: The language of this article should be polished to improve its readability. And one more thing, is Wulumuqi in the address of the corresponding author the right English name of the city? And the second address is not complete.**
Response:

Thank you for bringing this to our attention. We have thoroughly reviewed the language structure of the entire article and addressed numerous language-related issues, thus enhancing the English proficiency and readability of the manuscript, as exemplified in lines 37-38 and 63-63. We have conducted a thorough review of the entire manuscript and addressed numerous language issues, such as those in lines 37-38,39-44,63-64,65-68,104-105,116-117,216-217 307-308 ,237-238 and 412-414 among others (marked in the text; not listed here). Furthermore, Wulumuqi is a correct English name for the city, and it is more often written as Urumqi. Following your suggestion, we have completed the second address accordingly. Thank you very much for your suggestion.

We appreciate for Editors/Reviewers’ warm work earnestly, and hope that the correction will meet with approval.

Once again, thank you very much for your comments and suggestions.
